# Contact lens fitting and changes in the tear film dynamics: mathematical and computational models review

**DOI:** 10.1007/s00417-024-06400-5

**Published:** 2024-03-02

**Authors:** Darshan Ramasubramanian, José Luis Hernández-Verdejo, José Manuel López-Alonso

**Affiliations:** Faculty of Optics and Optometry, Complutense University of Madrid, Madrid, Spain

**Keywords:** Tear film, Contact lens, Biomechanics, Multiphysics

## Abstract

**Purpose:**

This review explores mathematical models, blinking characterization, and non-invasive techniques to enhance understanding and refine clinical interventions for ocular conditions, particularly for contact lens wear.

**Methods:**

The review evaluates mathematical models in tear film dynamics and their limitations, discusses contact lens wear models, and highlights computational mechanical models. It also explores computational techniques, customization of models based on individual blinking dynamics, and non-invasive diagnostic tools like high-speed cameras and advanced imaging technologies.

**Results:**

Mathematical models provide insights into tear film dynamics but face challenges due to simplifications. Contact lens wear models reveal complex ocular physiology and design aspects, aiding in lens development. Computational mechanical models explore eye biomechanics, often integrating tear film dynamics into a Multiphysics framework. While different computational techniques have their advantages and disadvantages, non-invasive tools like OCT and thermal imaging play a crucial role in customizing these Multiphysics models, particularly for contact lens wearers.

**Conclusion:**

Recent advancements in mathematical modeling and non-invasive tools have revolutionized ocular health research, enabling personalized approaches. The review underscores the importance of interdisciplinary exploration in the Multiphysics approach involving tear film dynamics and biomechanics for contact lens wearers, promoting advancements in eye care and broader ocular health research.


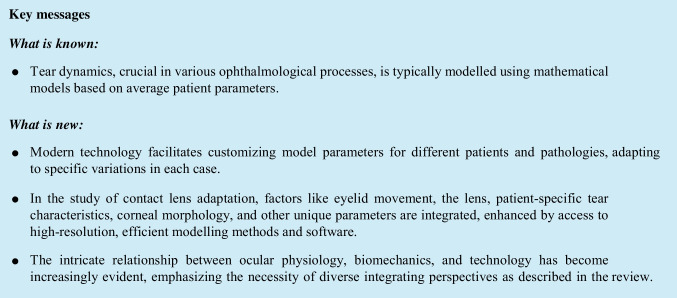


## Introduction

The choice and fitting of a contact lens (CL) involve considering various factors like lens material, mechanics, surface characteristics (like lubrication, friction, and wetness), design, corneal coverage, diameter, lens movement, base curve, tear exchange, and wear schedule (daily or continuous) [1]. All these factors including the subjective ones affect the general comfort of wearing the CL and have great variability between different people. Among these factors, one of the most important is the dynamics of the tear film as it conditions the movement of the lens with blinking and its friction on the eye [2, 3]. The tear film, in particular, serves as a critical interface between the CL and the corneal surface. An optimized tear film not only ensures lens comfort but also maximizes visual clarity and prevents complications often associated with lens wear.

**Fig. 1:**
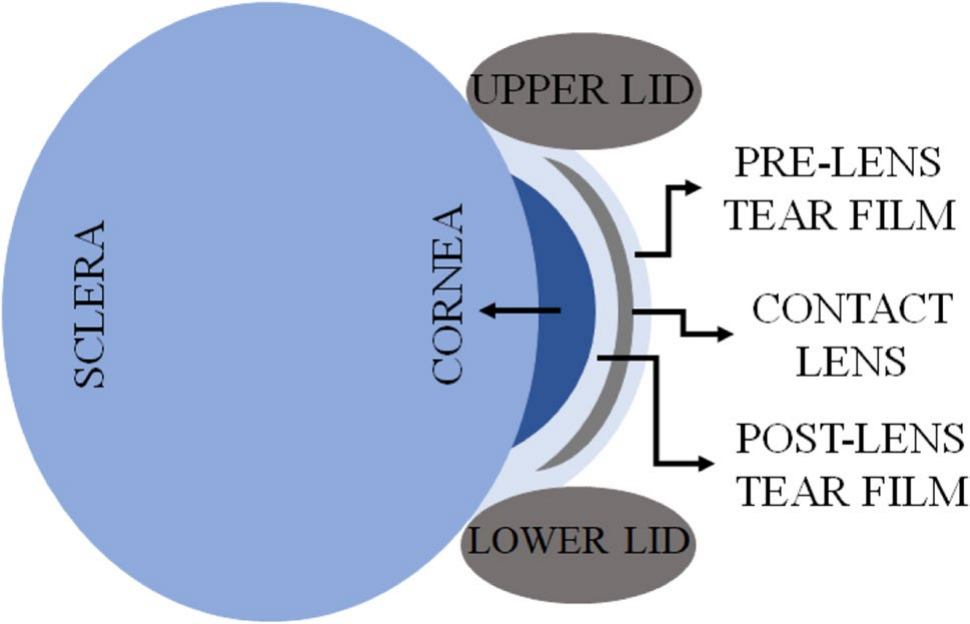
Schematic of the ocular surface highlighting the interaction of CL with PLTF and PoLTF layers, impacting stability

The human tear film, a focal point of extensive research, is pivotal for ocular health and visual acuity, affecting aspects like dry eye onset, CL interaction, and refractive quality. It is a multi-layered shield on the front of the cornea, providing defense against irritants and pathogens, lubricating the eye, supplying nutrients, facilitating smooth light refraction, protecting against foreign bodies, and aiding healing. Comprising a mucin layer near the cornea, a central aqueous layer, and an outer lipid layer [4], the tear film structure, with a total thickness of 2 to 5.5 µm [5], ensures stability. Each layer plays a crucial role, especially when a CL is used, dividing the pre-corneal tear film (PCTF) into pre-lens (PLTF) and post-lens (PoLTF) [6, 7]. The upper eyelid, acting as a “lid wiper,” spreads the tear film across the ocular surface during blinking [4]. Understanding these segments is key to appreciating the comfort and ocular surface health in CL wear, as they significantly alter tear film behavior and distribution, as shown in Fig. 1.

The tear film, vital for eye health, is traditionally viewed as having three layers: an inner mucin layer for even tear spread and friction reduction, critical in preventing dry eye disease (DED) [8]; a middle aqueous layer with water, electrolytes, and nutrients for clear vision and comfort [9]; and an outer oil-based lipid layer, produced by meibomian glands, to inhibit evaporation and maintain clarity [10]. Recent research, however, suggests a two-part model comprising the lipid layer and a combined mucin-aqueous layer [11], providing a more integrated perspective increasingly acknowledged in ocular studies. The question of why we study the tear film formed during each blink has intrigued various researchers. This complex and dynamic biological structure significantly influences our visual capabilities. Both theoretical and experimental model systems have the potential to advance our understanding of tear dynamics [12]. Numerous biologically based experimental models, ranging from various mammals to in vitro models utilizing components from these species and humans, have contributed to our comprehension of tear dynamics. Lid motion, a critical factor in maintaining an intact tear film on the ocular surface, lasts a mere 250 ms and can be captured with high-speed cameras [13].

The structure and function of the tear film are still not fully understood, with several factors contributing to DED [1, 14, 15]. DED, characterized by discomfort and potential vision impairment, has diverse origins, including insufficient tear production, increased evaporation, and imbalances in tear composition. External factors like prolonged screen use and environmental conditions, alongside internal factors like age, hormones, and medications, can exacerbate the condition. To effectively manage DED, it is crucial to go beyond the simplistic notion of inadequate tear production and consider the complex interplay of these factors. Clinically, tear breakup time (TBUT) is a pivotal metric, with reduced TBUT often indicating tear film instability, assessable through tests like the Schirmer test or anterior segment optical coherence tomography [16]. A comprehensive approach to DED management encompasses various strategies, including the use of artificial tears, prescription of anti-inflammatory medications, and lifestyle modifications [17]. Addressing the root causes and adopting a combination of these strategies ensures a comprehensive approach to treating DED.

This review is structured as follows: The second section provides an overview of mathematical models for simulating tear dynamics in interaction with CLs, subdivided into tear film dynamics and their incorporation into Multiphysics models encompassing eye and CL mechanics. These models require subject-specific characteristics, and recent technological advancements have expanded our ability to estimate and measure such characteristics, enhancing the adaptability of computational models for diverse subjects or populations. The third section reviews techniques for characterizing blinking (a key factor in CL movement) and other customizable parameters. The fourth section discusses the strengths and limitations of computational models, concluding with general insights.

## Mathematical models

This section focuses on mathematical models, which are crucial for understanding the complex interaction between CLs and the ocular surface, analyzing tear film response to blinking and simulating CL fitting mechanics.

### Tear film dynamics

Different mathematical models have been subject to prior research and were dedicated to helping study the dynamics of tears and TBUT [12, 18]. Most tear film models are 1D single-layer models simplifying with consideration of the aqueous layer to be a Newtonian fluid [12, 18–20] and treating the tear film lipid layer as an insoluble surfactant monolayer [21, 22]. Researchers assumed the shape of the human cornea is negligible, and theoretical articles suggest using Cartesian coordinates on a flat substrate to develop models for the tear film and it is referred to as flat cornea approximation [19, 23]. Models were simplified to investigate important effects on tear dynamics such as evaporation and gravity over the open surface of the eye [19], osmosis across the corneal surface [12], Marangoni effects induced by varying lipid concentration [21], and complete and partial blinks [20, 24], among others.

Expanding on the dynamics of tears and TBUT from various mathematical models, research highlights the crucial parameter of tear film thickness, offering detailed analyses of the PCTF, PLTF, PoLTF, and the lipid layer [6, 7]. Contrasting earlier measurements, recent investigations estimate the human PCTF thickness at about 3 μm, although the thickness can vary [7, 25]. Post-blink, the tear film is influenced by surface tension gradients, and its TBUT is closely related to the thickness of the lipid layer, with factors like surface tension and evaporation playing roles [18, 26–28]. Meanwhile, Wong et al. [18] provided insights into the deposition process of the tear film, noting that the exposed eye section of the coating measures approximately 10 μm. Their model predicts film thickness and post-blink lipid spreading, showing that the film quickly thins at the edges and breaks when it becomes too thin, a process influenced by tear viscosity, initial thickness, and observed TBUT.

Specific studies focusing on the physical properties of the tear film highlighted key factors like viscosity [29] and surface tension [30], crucial for understanding tear film behavior and stability on the ocular surface. It found that healthy eyes typically exhibit a tear viscosity of about 6 mPa-s, in contrast to the higher average of 30 mPa-s in dry eye conditions, suggesting that tear film rheology could be significant in diagnosing and managing ocular issues, particularly for CL wearers [29]. Furthermore, the research revealed that tears from dry eye patients have increased surface tension compared to those from healthy eyes, contributing to a reduced TBUT [31]. This heightened surface tension, coupled with increased viscosity, leads to greater tear film instability and quicker tear film breakage, exacerbating dry eye symptoms [30]. These findings emphasize the complexity of tear film dynamics and the interaction of several factors in causing ocular discomfort and visual disturbances.

An in-depth analysis of tear fluid characteristics reveals its non-Newtonian nature due to the presence of molecules like proteins, lipids, electrolytes, and mucins [9, 32]. These components exhibit shear-thinning behavior [29], impacting tear film dynamics when lipids are removed [33]. Some studies have neglected the influence of the corneal curvature, assuming a spherical substrate shape [34], while others explored cylindrical or prolate spheroid geometries [35, 36]. While the prolate spheroid approximates the human cornea, research suggests that corneal shape has minimal impact on tear film thinning rates, often leading to the assumption of a flat cornea in computational models of tear film dynamics [23]. Understanding tear fluid properties and substrate curvature helps refine tear film models.

**Fig. 2:**
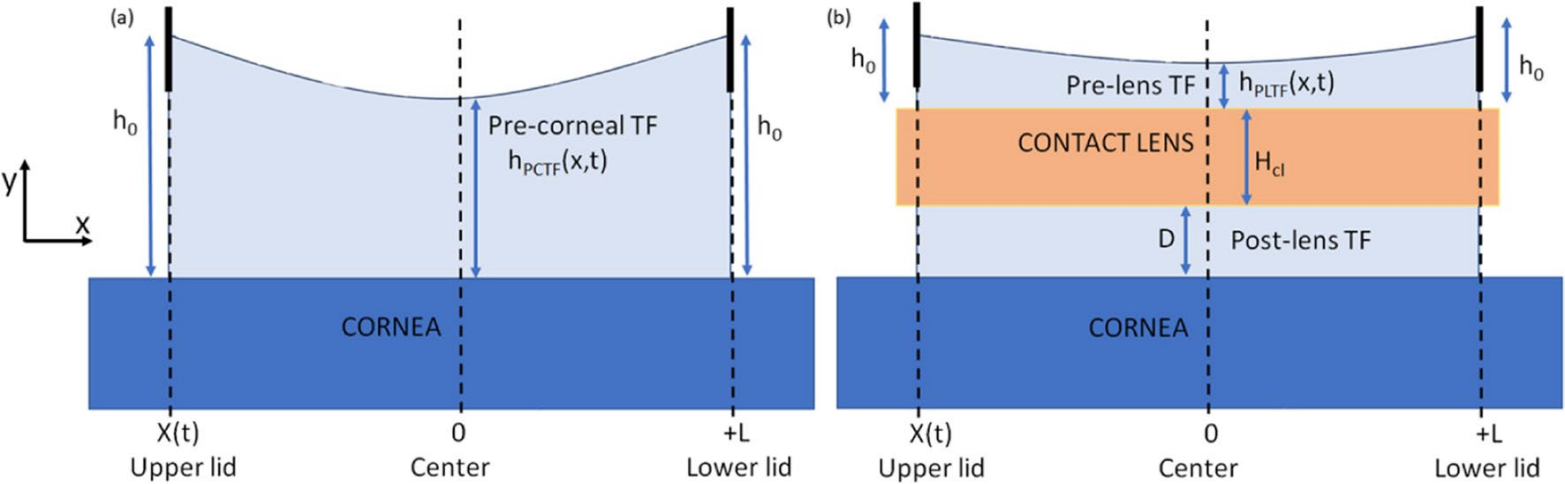
Schematic diagram from a mathematical viewpoint: (**a**) PCTF; (**b**) PLTF. (Parameters: X(t) – position as a function of time t; L – half-width palpebral fissure; h_0_ – initial tear meniscus height from both eyelids; H_cl_ – thickness of CL; D – PoLTF thickness; h_PCTF_, h_PLTF_ – PCTF thickness and PLTF thickness respectively as a function of time)

The exploration of tear film dynamics begins with an examination of parameters related to the ocular surface, emphasizing mathematical models that detail tear film formation and relaxation during blinking, as depicted in Fig. 2 [19]. These models, focusing on tear film thickness [7], delve into the evolution of the aqueous layer and consider factors like evaporation and heat transfer [19]. Typically, simulations begin with an initial condition, assuming uniform tear film deposition except for menisci near the eyelids. While many models simplify the lipid layer by assuming a stress-free upper surface [20, 22, 24], evidence suggests its role in particle movement [21, 37, 38]. Theoretical studies explore mathematical models with different blinking characteristics, incorporating upper lid movement during the opening phase of the eye [18, 22]. These models introduce fluxes to estimate tear supply, concluding that no-flux conditions fail to provide adequate coverage, necessitating tear fluid flux from the eyelid. Additionally, the impact of the lipid layer highlights altering the distribution of the film, influencing film height between blinks [18, 37]. These models also introduce critical concepts like the stress-free limit (SFL) and uniform stretching limit (USL), marking milestones in tear film research.

Another tear film model was devised to investigate tear film evolution across multiple blink cycles, focusing on the opening and closing of the eye. Lid movements were characterized using two approaches: sinusoidal motion [20] and realistic blinking [24]. Braun and King-Smith employed a sinusoidal blink model [20], defining each cycle as a period and identifying periodic solutions for both complete and partial blinks. This model revealed distinctions between the superior and inferior tear film layers and replicated in vivo tear film observations during partial blinks, offering insights into defining complete blinks based on fluid dynamics principles [20].

In contrast, the realistic blink model incorporated the entire blink cycle, including lid opening, open-eye duration, and closure, based on actual lid motion data [13, 39]. This approach introduced flux boundary conditions accounting for lacrimal gland supply and punctal drainage. Results indicated thicker tear film near the moving end during lid opening and closing, with thinning near the ends during stationary, fully open phases of the eye, providing a more comprehensive view of tear film dynamics [24]. Recent advances in imaging and mathematical models of tear film dynamics during blinking have deepened our understanding, with increased use of simultaneous imaging and improved OCT instruments promising further insights [40].

Mathematical models enhancing our understanding of tear film dynamics have concentrated on the lipid layer, particularly modeled as a polar lipid monolayer [22, 37]. These models demonstrate how concentration gradients induce Marangoni flow and affect evaporation rates within the tear film lipid layer, which is sensitive to variations in pressure, temperature, and surfactant concentration [41]. Although effective in capturing TBUT due to evaporation, these models struggle with identifying increased evaporation rates influenced by surfactant concentration. In scenarios considering both fully open and half-closed eye states, they compute high lipid concentrations near the lower lid during interblink, propelling the lipid upward and consequently dragging the aqueous tear fluid. When the eye half-blinks, this concentration peaks at the center of the eye, causing rapid tear film thinning. Another model examines the evolution of tear film thickness and lipid concentration during blinking [21], revealing that higher lipid concentrations amplify the Marangoni effect, driving lipids toward the upper lid. This model, framed as a coupled partial differential equation, shows that the presence of lipids not only thickens the tear film due to increased fluid flow but also results in a non-uniform lipid distribution across the tear film.

Tear film dynamics research has undergone significant evolution, introducing a model with a lipid reservoir continuously supplying lipids to the system and altering boundary conditions to control the impact of the lipid flow on tear film evolution [42]. Simultaneously, studies have explored rapid tear film thinning linked to uneven lipid layer distribution, emphasizing the correlation between a healthier, more uniform lipid layer and an extended TBUT due to lower surface tension [43]. Furthermore, investigations have utilized fluorescein imaging to simulate tear film thinning and solute transport, aligning simulated fluorescein intensity with in-vivo observations to differentiate between evaporative and tangential flow-driven tear thinning mechanisms. Prior research also qualitatively observed tear film thickness distributions and a drop in polar lipid content near the lids during blinking [21]. Additional model variations incorporated a dilute surfactant model and a thick aqueous layer with large menisci, revealing evidence of substantial lipid remnants after the upstroke of the blink cycle and the potential for a significant boundary thickness to facilitate tear film development during a full upstroke [38].

Briefly, the dynamics of the tear film are crucial for ocular health, and mathematical models provide invaluable insights into the layered complexity of the tear film. In the next section, we will explore computational solutions to the tear film dynamics in the presence of CLs, further advancing the knowledge in this field.

#### Presence of CLs

The interaction between the tear film and CLs is an intricate aspect of ocular physiology, as depicted in Fig. 2b, with implications for lens comfort and visual acuity. Investigating the dynamics of tear film behavior in the presence of CLs provides insights into the optimal design. Central to this exploration is the mathematical modeling of tear film behavior. Expanding the tear film models, recent advancements have incorporated specific parameters pertaining to CL wearers. Utilizing a lubrication theory-based approach, these models adeptly describe the dynamics of tear film in the context of blinking and CL wear [44–48]. It is worth noting that in the mentioned studies, the tear film is bifurcated into two distinct layers: the PLTF, which is the fluid layer sandwiched between the CL and the external environment, and the PoLTF, situated between the CL and the corneal surface.

Hayashi and Fatt [44] used lubrication theory to investigate tear exchange caused by the compression of a soft CL by the eyelid against the cornea, finding that each blink leads to an estimated 10–20% tear exchange with typical film thicknesses of 8 to 10 microns, highlighting the importance of blinking for maintaining tear film balance in CL wearers and the need for understanding tear dynamics to improve CL design and guidelines. Building on these findings, subsequent research delved into tear film dynamics with CLs, considering factors like lens thickness, permeability, gravitational effects, and slip models at the fluid-lens interface [45]. A complex mathematical model, derived by applying a lubrication approximation to hydrodynamic motion equations and considering the porous layer of the tear film, was developed to study the post-blink film evolution, revealing that increased lens thickness, permeability, and slip could accelerate film thinning, although these changes have minimal effect under standard CL conditions.

Dunn et al. [46] investigated the impact of blinking on tear film dynamics with soft hydrogel CLs, observing that blinking can either partition the tear film or fully integrate it into the CL, leading to relative sliding between the lens, corneal epithelium, and eyelid wiper. Their numerical model shed light on the pressures and sliding speeds involved, emphasizing that eyelid-lens interaction predominantly occurs in a hydrodynamic regime and is critical for understanding the lubrication behavior of CLs, particularly regarding ocular sliding, loading, and the potential for surface damage due to shear stress [2]. Building on these findings, Talbott et al. [47] explored the effects of evaporation on the PLTF with permeable CLs, noting how evaporation reduces PLTF thickness and leads to fluid loss through the lens. They employed lubrication theory to formulate an equation representing PLTF thickness, accounting for evaporation, thermal transfer, and capillary action. Their study compared comprehensive and simplified models, offering insights into fluid loss due to evaporation and contributing to a deeper understanding of the fluid dynamics involved in CL wear.

Anderson et al. [48] furthered the understanding of tear film dynamics with CLs by examining the partitioning of the PCTF similar to prior research, with thicknesses ranging from 1 to 5 µm, in contrast to the considerably thicker CLs (50–400 µm). They noted that CLs are subject to forces in both horizontal and vertical directions during blinking, with recent studies focusing more on vertical movement. Chauhan and Radke [49] evaluated this vertical motion using an innovative method based on mechanical force balance, considering forces from the eyelids, gravity, elasticity, and viscosity, and integrating parameters like lens attributes and tear film thickness variations. They discovered that the downward movement of the lens during a blink is 2–3 times greater than during the interblink phase, indicating that current testing methods may overlook significant aspects of lens movement. This research emphasizes the need for more comprehensive experimental approaches in understanding CL behavior and tear film dynamics.

Maki and Ross [50] introduced a novel method to calculate the suction pressure under a soft CL, focusing on how the lens deforms under the combined forces of the tear film and eyelid blink. Their findings revealed that with a consistent eye shape, the center of the lens experiences more suction pressure as the curvature radius of the lens increases, while peripheral pressure decreases, and negative pressure in the transition zone increases for larger radii. Building on this, one research [51] examined the impact of CL design on ocular health and how blinking affects lens adaptation, particularly how the lens attempts to regain its shape and generates suction in the PoLTF, influencing tear fluid movement and potential fluid exchange at the lens edge. Another study [52] employed a variational method to assess elastic stresses in CLs and their associated suction pressure, providing solutions to the Euler–Lagrange equation for lenses with consistent thickness, although challenges arise with variable lens thickness. These studies advance the understanding of the forces at play in tear film dynamics and CL behavior.

In summary, tear film dynamics with CLs merge biomechanics and fluid dynamics, with foundational research offering insights into tear film behavior, lens design impacts on ocular health, and underlying mathematical models. The next section will focus on the methods and challenges in solving these models, enhancing our understanding of the topic.

#### Numerical methods

Tear film dynamics involve creating non-linear partial differential equations with appropriate conditions, and MATLAB is a commonly used platform for solving these models [53]. MATLAB simplifies complex data analysis, offering a programming language for numerical computations and mathematical tasks with functions for matrices, algorithms, and user interfaces. Researchers employ MATLAB to solve mathematical models of tear film dynamics, often using the finite difference method [54], which is straightforward but may require a high number of grid points, making it time-consuming [19, 20, 22, 37, 38]. Despite its simplicity, this approach demands additional studies on stability and accuracy.

Alternatively, the Chebyshev spectral collocation method provides a more advanced solution [55]. It utilizes non-symmetric mapping to minimize point spacing, transforming equations into a time-dependent system of differential algebraic equations. This method further enhances accuracy and computation speed through a modified non-symmetric mapping and two input parameters, reducing errors, particularly in higher-order derivatives [56]. These techniques offer researchers powerful tools to analyze tear film dynamics effectively, balancing ease of use and computational efficiency [24, 48].

Concisely, the analysis of tear film dynamics using computational methods emphasizes the importance of mathematical models in predicting tear film behavior, leading to a subsequent section on the mechanical interplay between the cornea, CL, and ocular surface.

### Mechanical properties on CL-ocular surface interaction using computational finite element approach and software packages

The simulation of the computational mechanical models gives an understanding of the impact of the fitting of CL shape over the eye and their corneal pressure and friction. There are not many available models of this type in a Multiphysics approach due to the disparity in in-vivo measurement parameters and real characteristics of complex fluid dynamics of tear film [2, 46, 57, 58]. The finite element analysis of the cornea and CL has been studied in the past as a structural mechanism, with the assumption that the tear film is not considered.

Finite element models integrating the mechanical properties of the CL, cornea, and sclera, alongside their interaction with the eyelid, offer valuable insights into the structural mechanics of these components. This approach facilitates the analysis of stress and strain on the cornea and CL [46, 59, 60] and illuminates deformation patterns and frictional aspects of the CL and cornea [2, 58, 61]. Further enhancing our understanding, the modeling of the human eye in the realm of ocular biomechanics and physiology employs these finite element models to account for individual variations in eye shape. While initial models simplified the cornea and sclera as spherical surfaces with uniform thickness [62, 63], subsequent research has increasingly focused on the anatomical complexities, particularly the variable thickness of the cornea and sclera [64–67], providing a more nuanced and accurate representation of the structure of the eye.

The complex geometry of the human eye merits a more detailed examination. Within the model parameters, various studies define the sclera with an outer radius of 11.5 mm, while the cornea is characterized by an outer radius of 7.8 mm [67–69]. The cornea is neighbored by the limbus, which precedes three distinct segments, symbolizing various scleral sections [67]. Positioned next to the vitreous chamber is the retina, which is essential for vision. Angular metrics from the central axis to diverse points on the scleral and retinal formations elucidate the internal geometric interconnections of the eye. The retina, crucial for vision, lies next to the vitreous chamber. Insights into the internal geometric relationships of the eye are gleaned from angular measurements from the central axis to specific points on the scleral and retinal structures. Additionally, distances are referenced as outlined in the literature [67].

In the design of CL geometries, characteristics like thickness, base curve, diameter, and material properties were meticulously studied [48, 49, 62, 63, 70, 71]. In the methodology aimed at designing a tri-curve lens with distinct geometrical attributes [62, 63], the study employed specific tools to craft the CL surfaces, requiring exact element and nodal definitions. The design intricacies encompassed individualized considerations for both the front and back surfaces. The back surface underwent careful design to ensure an optimal fit, whereas the front surface was tailored to match the intended optical power. To maintain a specific orientation on the eye, ensuring stability and comfort, the study introduced a weighting factor to the front surface to accommodate prism ballast in the lens [62, 63]. The boundary thickness between the transient zone and the peripheral zone was adjusted to enhance thickness in the lower meridians, guided by the weighting factor.

This section presents computational modeling focused on ocular mechanics, laying the groundwork for subsequent exploration of material models related to eye components and CLs in the following subsection.

#### Material properties

This subsection explores the detailed material properties of ocular components and CLs, which are crucial for enhancing vision correction and driving innovations in ophthalmology and CL design. CLs are typically modeled as Neo-Hookean materials based on parameters like Young’s modulus and Poisson’s ratio [62, 63].

Understanding the material properties of the sclera is vital in ocular biomechanics, leading to its diverse modeling in biomechanical literature. The Neo-Hookean model [67, 72, 73], often used to represent the sclera, treats it as a hyperelastic material capable of significant deformations, aptly reflecting the non-linear stress–strain relationship of soft biological tissues. This model is popular for its computational efficiency and biomechanical accuracy in evaluating the mechanical behavior of the sclera under various loads. Meanwhile, some studies employ the Ogden model [67, 72, 73] to describe the sclera, viewing it as a non-linear hyperelastic material. This model is particularly adept at capturing complex, anisotropic, and non-linear characteristics, making it suitable for detailed biomechanical analyses of scleral response under multifaceted loading conditions. In contrast, the sclera is sometimes simplified as a rigid body [74, 75], a useful approximation in scenarios where its deformation is not of primary concern, implying it remains undeformable regardless of external forces.

In modeling the cornea, various mechanical models are employed to capture its unique complexities. The Neo-Hookean model [67, 73, 76, 77] approaches the cornea as a hyperelastic material, ideal for simulating large deformations and addressing the non-linear stress–strain characteristics of soft tissues, balancing computational ease and biomechanical accuracy. Conversely, the Ogden model [73, 78, 79] provides a detailed representation of the stress–strain relationship under extensive strains, making it invaluable for complex biomechanical analyses. The Mooney-Rivlin model [65, 80, 81] extends this by introducing additional parameters for an enhanced depiction of the non-linear and anisotropic properties of the cornea. Additionally, the anisotropic, hyperelastic large-deformation constitutive model [65, 80, 82, 83] focuses on the collagen fiber orientation of the cornea, which is vital for refractive surgery. This model adeptly demonstrates the biomechanics of the cornea, influenced by age, hydration, and collagen alignment, transitioning from organized central patterns to random peripheral arrangements, offering a detailed view of corneal biomechanics [65, 80, 82, 83].

Having explored the detailed material models of ocular components, our focus now shifts to the computational techniques employed, elucidating how these material models are seamlessly integrated into finite element software for precise and effective analyses.

#### Computational methods and software packages

Finite element software such as FEBio [84] and Ansys [85] are pivotal for defining precise geometries and evaluating subtle changes in biomechanics and engineering, crucial for analyzing stress and displacement under various pressures [86]. These tools enable detailed Multiphysics simulations, as exemplified by COMSOL Multiphysics [87], which effectively couples physical phenomena like eye and eyelid mechanics with tear fluid dynamics, integral for simulating tear film behavior. FEBio specializes in finite element analysis of biological tissues, particularly soft tissues, while ANSYS provides extensive capabilities in finite element analysis, computational fluid dynamics, and Multiphysics, suitable for complex designs and product development. COMSOL Multiphysics, recognized for its ability to model intricate systems such as tear film dynamics, merges precision with ease of use, making it a valuable resource in diverse fields including engineering, industry, and academia [88].

## Customizing models with non-invasive techniques for subjects

Creating accurate CL models for optimal patient comfort necessitates integrating key parameters like tear properties, materials, and blinking dynamics. These parameters are categorized in Table 1 into three groups: physical parameters (tear film properties and eye biomechanics), customization parameters (quantifiable variables used as inputs to reflect individual differences), and CL-specific parameters (design and material aspects). Subsequent sections detail studies and tools for measuring subject-specific parameters, with metrics like TBUT validating model results against actual data.

**Table 1:** Parameters that influence the patient comfort and the CLs

Physical properties	Customization parameters	CL parameters
Viscosity	Tear meniscus	Base curve
Surface tension	Blinking speed	Thickness
Tear film density	Half-width palpebral fissure	Diameter
Material properties of cornea	Cornea radius of curvature	Elastic modulus
Material properties of sclera	Sclera radius of curvature	Poisson ratio
		Density

### Blinking characterization

Conducting experiments on blinking is crucial for refining mathematical models, advancing our knowledge of eye physiology, and enhancing interventions like CL design. Simulation models of tear film dynamics also incorporate the kinematics of blinking, which can vary with age, between individuals, and due to CL use. Characterizing eyelid movement is especially important because it significantly influences CL movement and, consequently, tear dynamics. The section aims to comprehensively review techniques and findings related to eyelid kinematics, integrating them into customized tear film models for a personalized understanding of unique tear film behaviors and enhancing CL design and eye care interventions.

**Fig. 3:**
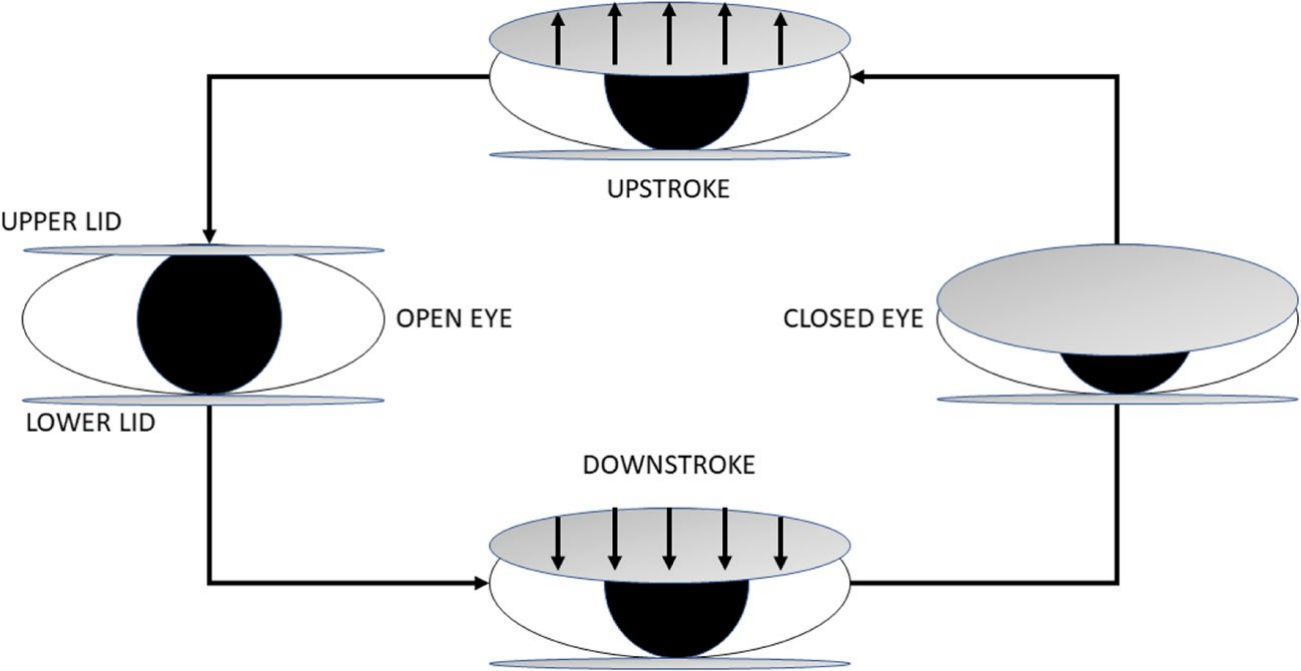
Illustration of the four phases of the blink cycle, focusing on upper eyelid movement. Per [40], most blinks are partial, with “closed eye” as a “turning point,” not full closure

Blinking, a semi-autonomous eyelid movement, is essential for spreading tears, lubrication, and tear drainage. The blink cycle involves four stages (Fig. 3) [40]: downstroke (closing phase), a point where the upper eyelid turns without touching the lower eyelid (eye closed or turning point), upstroke (opening phase), and the upper eyelid returning to its uppermost position (eye open). Research indicates many blinks do not fully close, which is crucial for understanding blink dynamics and variations [89]. Furthermore, in vivo studies recording post-blink tear film particle movement suggest that surface tension differences [34], driven by varying concentrations of surface-active materials, likely facilitate tear film movement toward the upper lid. This “surface tension gradient mechanism,” supported by surface chemistry data and hydrodynamic equation approximations, aligns with experimental findings, elucidating crucial aspects of tear film dynamics post-blink.

Exploring tear film dynamics with blinking, Owens and Philips [90] focused on tear-spreading post-blink in healthy individuals. They utilized video recordings to track particles in the tear film, uncovering that tears move upward over the cornea at a velocity of 7.34 ± 2.73 mm/s, stabilizing within approximately 1.05 ± 0.30 s. This velocity and stabilization time, influenced by factors like meibomian glands and irritants, offer key metrics for non-invasive tear assessments. In subsequent research [91], they examined tear film functionality challenges, particularly in the context of CL wear and dry eye management. They observed tear dispersion over the cornea at around 10 mm/s post-blink, highlighting the impact of eyelid velocity, tear meniscus, viscosity, and surface tension on tear-spreading rates. Complementing this research, Berke and Mueller [13] developed a mathematical model to simulate lid motion, incorporating parameters such as fissure width and blink duration. Their model, aligning with published data [39], provides theoretical predictions on lid velocities, offering a more comprehensive understanding of blinking dynamics (Fig. 3).

In our exploration of tear film dynamics, we delve into the intricacies of upper lid movement over time, often derived from high-speed digital camera recordings capturing blinks at 60 frames per second or more [92–97]. Utilizing this data, we construct a straightforward two-parameter continuous curve to represent the vertical velocity of the upper lid, enabling numerical simulations rooted in real-world observations. Tear film dynamics during the upstroke of the blink cycle and the subsequent open phase are seamlessly integrated with blinking data and fitted into an exponential function over time [22, 37]. An extension to the blinking model introduces a sinusoidal function [20], addressing both upstroke and downstroke within the blink cycle and providing solutions for multiple blink cycles without thickness evolution in the open phase for complete and partial blinks. This theory reveals that partial lid closure can yield periodic solutions. Another model incorporates realistic lid motion, aligning with observed data [13, 24, 39], and enhances tear thickness measurements for both the PCTF and PLTF. Previous models also consider multiple blink cycles, assuming an initial tear meniscus height before the first blink and adjusting it for subsequent blinks, a critical consideration for CL wearers [48]. CL use alters tear film thickness across various blinks, potentially impacting overall blinking dynamics. A comprehensive understanding of blinking dynamics offers valuable insights into CL-related alterations in tear film behavior.

Analyzing blinking characteristics, including blink amplitude, duration, and peak speed, offers a precise examination of blinks. High-speed cameras at 600 frames per second have been utilized to study fast voluntary eye blinks [92], revealing asymmetric motion with durations of about 500–600 ms and average peak speeds of approximately 150–250 mm/s during eye-opening and 80–160 mm/s during closure. This approach provides highly accurate results for investigating blink kinematics [92, 94]. Blinking characterization has also been explored through physical magnitudes related to muscle action, with eyelid position correlated to variations in reflected light. Physiological phenomena, their derivatives, and products are used to define blink features, including power, work, and impulse. These advancements facilitate the development of biometric identification systems based on physical or physiological characteristics, where evaluated parameters can predict eye and CL pressures [93].

Prior research has addressed CL pressures, primarily considering tear fluid surface tension and eyelid-induced pressure. Tear fluid surface tension, measured via Wilhelmy balance [98], ranges from 46.6 ± 3.8 to 71.5 ± 1.3 mN/m. However, the typical value of the tear fluid aligns closer to the lower range of 46.6 mN/m across ages [34]. Eyelid pressure on the cornea ranges from 1 to 5 kPa, while CL wearers experience 12–18 kPa [61, 99]. These pressures are integrated into finite element analysis [100], modeling the eye and CL as a complex tribological system, aiding in predicting their behavior under various conditions. An intraocular pressure of 10–21 mmHg is additionally applied to the bottom surface of the cornea to simulate eye fluid pressure [101].

Digital particle image velocimetry (DPIV) is a non-intrusive analytical technique commonly used for quantitative flow mapping, particularly for particle tracking [102]. The accuracy of flow measurements in DPIV depends on various computational elements, including image pre-processing, sub-pixel peak estimation, data validation, interpolation, and smoothing methods. PIVlab, an open-source DPIV analysis tool [102], is freely available as a MATLAB toolbox [103, 104], offering a user-friendly graphical interface. It has been adapted for spatially characterizing eyelid movements [96] in MATLAB [53]. The experiment involves capturing a series of high-speed camera images during a blink sequence. These images are then analyzed using a cross-correlation method to establish velocity maps for interrogation windows in each frame. Calibration is done using a reference distance, and the *x* and *y* components of velocity in m/s are exported for further analysis, which can be easily imported into MATLAB or other software packages. This approach provides valuable insights into individual blinking behaviors aligning with the findings discussed in previous sections regarding blinking characteristics [13, 90–92].

In summary, upper lid motion significantly impacts tear film dynamics, explored through various experimental and theoretical approaches. The next section will delve into additional customizable parameters for optimizing comfort in CL wearers.

### Model parameter customization

Customizing mathematical models to reflect individual ocular conditions is crucial due to the variability in physiological parameters like tear film volume, meniscus height, eyelid distance, and corneal curvature. Accurate, patient-specific data are vital for these models to accurately represent the biomechanical and biophysical behaviors of the eye. This necessitates the use of non-invasive diagnostic techniques, which are evolving to capture detailed eye anatomy and function without patient discomfort. Integrating data from these advanced methods allows for refining mathematical models to simulate individual tear film dynamics more precisely.

Optical coherence tomography (OCT) is a technique developed and used to visualize structures arranged in layers. OCT has evolved into various commercial systems. Time domain OCT measures backscattered light length by moving the reference mirror, while frequency domain OCT, including swept-source and spectral domain methods, uses broadband interference [105]. Anterior segment OCT [106], a type of Fourier domain OCT, offers a comfortable and objective means of measuring tear meniscus parameters, aiding in early dry eye detection. While it measures tear film characteristics, it may not reveal significant differences between dry eyes and healthy individuals despite hyperosmolarity from increased tear film evaporation [107]. High-resolution OCT equipment has been used for detailed tear film dynamics visualization and lens fitting [108], but such equipment is not always readily available. Most commercial OCT systems can easily measure the tear meniscus [109–112], a valuable parameter for characterizing dry eye or CL wearers and customizing mathematical tear film models.

Non-invasive corneal topography diagnoses conditions like keratoconus and astigmatism and evaluates refractive surgery outcomes by detailing anterior corneal curvature and shape [113]. The pentacam topography employs a rotating camera to capture 3D images of the cornea [114], offering insights into parameters such as anterior and posterior corneal contour, elevation, pachymetry, and astigmatism. Alongside corneal assessment, the Oculus Keratograph 5 M serves as another valuable non-invasive tool [115]. It analyzes tear film dynamics, providing essential information such as TBUT, which helps distinguish between dry eye and stable tear film conditions. Utilizing white or infrared illumination, this device evaluates tear film stability, non-invasive TBUT, tear meniscus height, and the lipid layer, contributing to a comprehensive understanding of ocular health and tear film behavior.

Non-invasive techniques for measuring TBUT, which avoid contact with the eyelids, are gaining preference for their accuracy and consistency compared to invasive methods [116]. The non-invasive TBUT (NIBUT) test, often conducted using advanced instruments like keratography, offers a more feasible and accessible approach for detecting DED, demonstrating superior diagnostic performance. Additionally, thermal imaging cameras employing infrared thermography capture the ocular surface temperature and its distribution, producing thermogram images [117]. This method tracks temperature changes on the cornea and eye surface post-blink, where heat exchange from the lower lid resets the surface temperature, gradually cooling as the eye remains open. This technique is instrumental in assessing tear film stability and monitoring tear fluid evaporation during and after blinking, providing valuable insights into ocular health [118].

## Discussion

Understanding the intricate structure and functionality of the tear film is essential for effectively managing conditions like DE [1, 14, 15], which can significantly impact visual acuity and overall ocular health. This review aims to explore mathematical models, blinking characterization, and non-invasive techniques to enhance our knowledge and refine clinical interventions for ocular conditions [4, 5, 8–10].

Mathematical models have played a crucial role in unraveling tear film dynamics on the ocular surface, shedding light on its formation, post-blink relaxation, and factors like evaporation and heat transfer [19, 41, 118]. While these models provide valuable insights, they often face limitations due to simplifications, such as neglecting the effects of the lipid layer. Incorporating realistic blinking patterns and conducting evaluations over multiple cycles can improve the representation of tear film dynamics [20, 22, 24], but challenges persist, particularly concerning surfactant-driven evaporation rates [10, 21, 37, 38, 42]. Ongoing research and the integration of advanced imaging techniques are pivotal for a more comprehensive understanding of tear film behavior and continuous enhancements in ocular surface studies.

Mathematical modeling of tear film dynamics in CL wear has revealed complex aspects of ocular physiology and lens design. These models effectively map the behavior of PLTF and PoLTF [7], considering parameters like lens thickness and permeability and capturing the intricate effects of blinking and evaporation [44–48]. However, the reliance on lubrication approximations in PLTF dynamics studies [45] and simplifications in models like those of Dunn et al. [46] and Talbott et al. [47] indicate certain limitations. Research by Chauhan and Radke [49], Anderson et al. [48], and Maki and Ross [50–52] has enriched our understanding of lens movements and forces despite challenges in testing methodologies and issues with varying lens thickness. Overall, these studies, while acknowledging their constraints, provide valuable insights, guiding future research toward refining lens designs and improving the CL-wearing experience.

Computational mechanical models, enriched by detailed analysis of materials in ocular components like the sclera and cornea, provide a profound understanding of eye biomechanics and CL fitting [64–67, 88]. These models illuminate the structural mechanics of the eye, capturing stress, strain, and deformation patterns in interactions involving the CL, cornea, sclera, and eyelid [2, 46, 59, 60]. While the Neo-Hookean model balances computational simplicity with biomechanical accuracy [62, 63, 67, 72, 73, 76, 77], the Ogden [73, 78, 79] and Mooney-Rivlin [65, 80, 81] models explore the anisotropic and non-linear behaviors of the sclera and cornea. Despite their effectiveness, challenges persist, including discrepancies between in-vivo measurements and model outcomes and oversimplifications in eye geometry or tear film representation. However, the integration of structural mechanics with tear film dynamics in a Multiphysics approach marks significant progress in ocular biomechanics research [62, 63]. The diversity of these models, considering their design and complexities, underscores the critical need for selecting appropriate models based on specific research goals and details in ophthalmological research and CL design [64–67, 88].

Exploring computational techniques for analyzing tear film dynamics, we find both strengths and weaknesses. MATLAB excels in data manipulation [53], in which the finite difference method [54], despite its simplicity, demands time and accuracy. The Chebyshev spectral collocation method enhances speed and precision in non-symmetric mapping [55, 56]. Software like FEBio is adept at simulating biological tissues [84], ANSYS offers comprehensive engineering solutions [85], and COMSOL Multiphysics is known for quick results and ease of use [87]. The choice of technique depends on the research goals and required detail.

Theoretically, models can be customized to individual blinking dynamics, improving comfort for CL users [40]. This area benefits from diverse studies and methodologies, including high-speed cameras and digital particle image velocimetry [96, 102], enhancing our understanding of factors like eyelid velocity and tear film stabilization [90–94]. Tools like PIVlab [102] on MATLAB allow dynamic, personalized analysis. However, the breadth of information can be overwhelming, making it challenging to discern key insights from minor details.

Ophthalmology has seen significant advancements in non-invasive techniques for eye assessment and diagnosis. Customizing mathematical models for each patient based on parameters like tear volume and corneal curvature enables personalized treatments. Technologies such as OCT, anterior segment OCT, corneal topography, and oculus keratograph 5 M offer in-depth insights into various eye conditions while ensuring patient comfort [105–107, 113–116]. Thermal imaging further enriches our understanding of tear film dynamics [117]. The continual integration of these advanced techniques promises to enhance patient care, diagnosis, and treatment, reflecting the dedication of researchers and clinicians to optimal eye health.

## Conclusion

In recent years, rapid advancements in both mathematical modeling techniques and the accompanying hardware have democratized the use of simulation models in research, industry, and ocular health institutions. These models prove invaluable when assessing multifaceted phenomena, as they allow for the incorporation of numerous complex factors. A prime example is the study of CL adaptation, where various variables such as lens properties, ocular surface morphology, blink dynamics, tear film characteristics, and their interactions with ocular elements converge. Furthermore, modern technology facilitates the measurement of crucial model parameters, enabling customization for different populations and individuals. In summary, the constructive collaboration between mathematical models and non-invasive tools in tear film dynamics research emphasizes the necessity of personalized approaches in ophthalmology. This interdisciplinary exploration sheds light on the intricate connections among ocular physiology, biomechanics, and technology, propelling advancements in CL wearer care and broader ocular health research.
